# Electrospun NiPd Nanoparticles Supported on Polymer Membrane Nanofibers as an Efficient Catalyst for NaBH_4_ Dehydrogenation

**DOI:** 10.3390/polym15051083

**Published:** 2023-02-21

**Authors:** Nasser Zouli, Ibrahim M. Maafa, Ahmed Abutaleb, Ayman Yousef, M. M. El-Halwany

**Affiliations:** 1 Department of Chemical Engineering, College of Engineering, Jazan University, Jazan 45142, Saudi Arabia; 2 Department of Mathematics and Physics Engineering, College of Engineering in Matteria, Helwan University, Cairo 11718, Egypt; 3 Department of Mathematics and Physics Engineering, College of Engineering, Mansoura University, El-Mansoura 35516, Egypt

**Keywords:** electrospinning, NiPd, membranes, H_2_, NaBH_4_

## Abstract

Sodium borohydride (SBH) hydrolysis in the presence of cheap and efficient catalysts has been proposed as a safe and efficient method for generating clean hydrogen energy for use in portable applications. In this work, we synthesized bimetallic NiPd nanoparticles (NPs) supported on poly(vinylidene fluoride-co-hexafluoropropylene) nanofibers (PVDF-HFP NFs) via the electrospinning approach and reported an in-situ reduction procedure of the NPs being prepared by alloying Ni and Pd with varying Pd percentages. The physicochemical characterization provided evidence for the development of a NiPd@PVDF-HFP NFs membrane. The bimetallic hybrid NF membranes exhibited higher H_2_ production as compared to Ni@PVDF-HFP and Pd@PVDF-HFP counterparts. This may be due to the synergistic effect of binary components. The bimetallic Ni_1−x_Pd_x_(x = 0, 0.05, 0.1, 0.15, 0.2, 0.25, 0.3)@PVDF-HFP nanofiber membranes exhibit composition-dependent catalysis, in which Ni_75_Pd_25_@PVDF-HFP NF membranes demonstrate the best catalytic activity. The full H_2_ generation volumes (118 mL) were obtained at a temperature of 298 K and times 16, 22, 34 and 42 min for 250, 200, 150, and 100 mg dosages of Ni_75_Pd_25_@PVDF-HFP, respectively, in the presence of 1 mmol SBH. Hydrolysis utilizing Ni_75_Pd_25_@PVDF-HFP was shown to be first order with respect to Ni_75_Pd_25_@PVDF-HFP amount and zero order with respect to the [NaBH_4_] in a kinetics study. The reaction time of H_2_ production was reduced as the reaction temperature increased, with 118 mL of H_2_ being produced in 14, 20, 32 and 42 min at 328, 318, 308 and 298 K, respectively. The values of the three thermodynamic parameters, activation energy, enthalpy, and entropy, were determined toward being 31.43 kJ mol^−1^, 28.82 kJ mol^−1^, and 0.057 kJ mol^−1^ K^−1^, respectively. It is simple to separate and reuse the synthesized membrane, which facilitates their implementation in H_2_ energy systems.

## 1. Introduction

Hydrogen (H_2_) is one of the promising renewable energy sources because of its various benefits, including zero emissions and a high energy density [1]. Nonetheless, a significant barrier thwarts its widespread use: the current limitations on safe and efficient hydrogen transportation and storage methods. The most common ways for storing and transporting hydrogen is in the form of high-pressure gas or liquid hydrogen [2,3]. However, these procedures need sophisticated equipment, due to which there has been a recent surge in research interest in synthesizing chemical hydrogen-storage materials that can safely and effectively store H_2_, such as ammonia borane (NH_3_BH_3_) [4,5,6], magnesium-based composite hydrides (MgH_2_) [7,8], and sodium borohydride (SBH, NaBH_4_) [9,10,11]. In the same context, SBH has also been studied extensively owing to its high hydrogen-storage capacity, controlled hydrogen release, and the high purity of produced H_2_ [6,7]. With a large hydrogen-storage capacity, 1 mol SBH hydrolyzes to 4 mol of H_2_ with 2 moles being produced from SBH and the other 2 moles from water, as shown in the below equation:

(1) $${\mathrm{NaBH}}_{4}+2H_{2}O\overset{\mathrm{catalyst}}{\to }4H_{2}+{\mathrm{NaBO}}_{2}$$

Self-hydrolysis of NaBH_4_ is exceedingly slow. Therefore, effective catalysts are needed to speed up the hydrolysis of NaBH_4_ and release hydrogen at a faster rate [12]. Precious-metal catalysts, such as Ru [12,13], Pt [14], and Pd [15,16,17,18,19], have the greatest catalytic performance in SBH hydrolysis. However, the applications of these catalysts are limited because they are rare and expensive [4,20]. Thus, transition-metal-based catalysts have been explored instead because they are cheap and available in abundance. Therefore, there has been a growing scientific interest in the development of catalysts based on abundant metals such as those based on Co [1], Ni [21,22], and Cu [23,24,25], which have been developed for SBH hydrolysis. However, they have shown only a moderate catalytic activity and poor long-term stability. To overcome these problems, an approach has been proposed which can simultaneously provide good catalytic performance and reduced cost by using the following two strategies: (1) producing highly active composite metal by exploiting the interaction of electronic and lattice effects in alloy catalysts [16,26,27,28], and (2) increasing the specific surface area and the number of active sites that could be accomplished by modifying the geometric features of catalysts [27,29]. Furthermore, it is difficult to activate water molecules with a single transition metal, and its catalytic activity is often lower than that of precious metal catalysts, making it less effective in splitting O-H bonds [26,29]. A combination of a transition metal and a precious metal is an excellent option for water splitting. Several nano-bimetallic catalysts have been developed which demonstrated improved catalytic activity in the dehydrogenation of SBH [30,31,32,33,34,35]. The catalysts have been prepared either by pyrolysis or the chemical reduction process [31,32,33,34,35]. However, poor particle-size distribution is a common problem with these catalysts because of the aggregation that occurs during preparation. Using catalysts improves the metal-specific surface area and catalytic stability/activity while retaining the metals in nanoscale. This might be a suitable strategy for enhancing the activities of the metals and decreasing the tendency towards the severe agglomeration of the metal particles during catalysis, without reducing the efficiency [36,37]. In addition, since the carrier can be readily separated, the catalyst may be recycled for further use. As a result, selecting an appropriate support is critical for enhancing catalytic performance and reuse efficiency. Consequently, a variety of support matrix materials, such as polymers [36,37,38,39,40,41], zeolite [42,43,44,45,46], nano-carbon [47,48,49], TiO_2_ [4,21,50,51], and Al_2_O_3_ [52,53,54,55,56,57] have been used to act as preservatives or as a sustaining matrix for the metal NPs in H_2_ generation from materials. Under varied reaction conditions, a polymer support containing metal NPs can demonstrate a synergistic influence on useful functionality, solvent tolerance, and lifetime [26]. The large specific surface area of fiber-shaped materials makes them well-suited for filtering and recycling applications [16]. The aim of this study is to prepare Ni_1−x_Pd_x_ (x = 0, 0.05, 0.1, 0.15, 0.2, 0.25, and 0.30)-PVDF-HFP NF membranes as a bimetallic catalyst for the dehydrogenation of SBH. Electrospinning was used to create the membranes, and then they were reduced chemically to obtain the final metallic catalyst. Electrospun catalysts containing bimetallic Ni_1−x_Pd_x_ NPs supported by a PVDF-HFP membrane were successfully fabricated, as evidenced by their characterization. Superior catalytic performance in H_2_ production from SBH was shown by the synthesized NFs. Catalytic activity was more for the prepared bimetallic Ni_75_Pd_25_. In addition, after seven cycles of reuse, this combination retained its stability. These hybrid catalytic-membrane NFs catalysts are promising candidates for hydrogen production because of their unique, efficient, and recyclable features.

## 2. Experimental Details

### 2.1. Materials

All of the chemicals used in this study were acquired from Aldrich Co., St. Louis, MO, USA: sodium borohydride (NaBH_4_, SBH, 98%), palladium (II) acetate (PdAc, 99.9%), N, N-dimethylformamide (DMF, 99.8%), acetone (99.5%), nickel (II) acetate tetrahydrate (NiAc, 98%), and PVDF-HFP (MW = 65,000 g/mol).

### 2.2. Experimental Work

Initially, a 15% PVDF-HFP solution was made by dissolving polymer powder in DMF and acetone in a ratio of 4:1. After that, a distinct composition of PdAc and NiAc was obtained by adding varying amounts of Pd salt into an aqueous solution of Ni salt in order to produce Ni_1−x_Pd_x_ (x = 0, 0.05, 0.1, 0.15, 0.2, 0.25, and 0.30). The mixtures were stirred for five hours to assure that Pd salt was completely dissolved. In separate glass bottles, the different solutions, each with its own distinct composition, were mixed with PVDF-HFP. The solutions were stirred at a temperature of 50 °C for 5 h to obtain homogenous sol-gels. To produce hybrid NF membranes, each sol-gel was loaded onto a lab scale electrospinner using a disposable plastic syringe. Copper wire that was immersed along the syringe from the back to front was connected to positive electrode of high-voltage power supply. At the same time, the negative electrode was connected with a rotating drum made of stainless steel and covered with aluminum foil. High voltage of 18 kV was applied in the 18 cm gap between the syringe and drum. After that, the sol-gel in the syringe was completely injected. The collected electrospun NF mats were wrapped in the aluminum foil, detached and dried overnight at a temperature of 30 °C under vacuum.

### 2.3. Chemical Reduction of Electrospun Nanofiber Mats

Pieces of NiPd@PVDF-HFP, each having a distinct composition, were placed in each specific beaker that contained 100 mL of methanol solution. The weight of each of the NiPd@PVDF-HFP pieces with different compositions was same. They were agitated for 30 min at room temperature. Then, NaBH_4_ was added gradually while stirring, and the mixture was mixed at room temperature until all of the bubbles had vanished. Particularly, the metal ions/NaBH_4_ molar ratio was set at 1:5 in order to achieve a complete reduction process. When the membranes were affixed to NaBH_4_, they immediately changed to black. After being removed from the NaBH_4_ solution, the membranes were rinsed with deionized water and dried in an oven set at 50 °C.

### 2.4. Characterization

The as-prepared catalysts were analyzed using techniques mentioned in our report [58].

### 2.5. NaBH_4_ Hydrolysis Using Prepared Catalysts

NiPd@PVDF-HFP NF-membrane catalysts containing varying ratios of Ni to Pd were loaded in a reactor consisting of two-neck flask. The flask was then tightly sealed before placing it into a thermostatically controlled water bath set at 25 °C. The reaction of catalytic hydrolysis was carried out at a temperature of 25 °C with 50 mL of 1 mmol SBH and 100 mg of catalyst. The solution was mixed by stirring at 1000 rpm. Hydrogen-gas production was calculated using the water-displacement approach. This allowed calculation of volume of gas. The evolution of gas occurred instantaneously, and the progress of the reaction was governed by the volume of H_2_ evolved, which can be measured by the displaced H_2_O from the burette at periodic durations of time of 120 s. The linear relationship of the volume of hydrogen with the function of time was obtained for the first 40 min, to determine the rate of hydrogen production (k). Additionally, the activation energy needed for the reaction was determined by measuring the volume of H_2_ produced at several temperatures ranging from 25 to 55 °C with 1 mmol SBH, and 100 mg catalyst. The test was also carried out at a constant temperature of 25 °C with varying amounts of catalysts (100, 150, 200, and 250 mg), as well as varying concentrations of SBH (1, 2, 3 and 4 mmol). During the recycling process, the lifetime of the newly developed membranes was also tested. This procedure was carried out for all the cycles with the same catalytic NFs membrane, so that the effectiveness of the catalyst could be measured. The temperature in the reactor was kept constant at 25 °C during each cycle, and 1 mmol of NaBH_4_ was injected into the flask in every cycle.

## 3. Results and Discussion

### 3.1. Hybrid-Membrane Characterization

**Figure 1:**
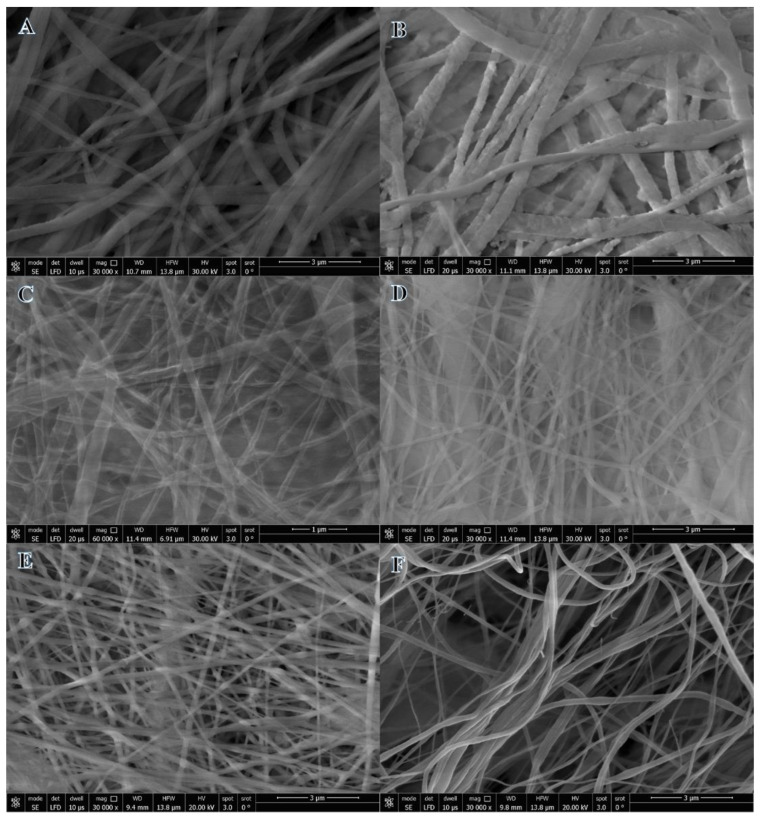
SEM images of PVDF-HFP (**A**), Ni@PVDF-HFP (**B**), Ni_95_Pd_5_@PVDF-HFP (**C**), Ni_90_Pd_10_@PVDF-HFP (**D**), Ni_85_Pd_15_@PVDF-HFP (**E**), and Ni_75_Pd_25_@PVDF-HFP NFs membrane (**F**).

PVDF-HFP membranes have recently been developed for a wide range of uses [59,60,61,62,63,64,65]. SEM images of nanofibrous PVDF-HFP membranes demonstrated the formation of an efficient nanofibrous structure which was free from beads (Figure 1A). It is well-established that the electrospinning process results in the synthesis of nanoporous-structure NFs. During electrospinning, solvents, most notably acetone, quickly evaporate, leaving nanopores in the electrospun NFs. The formation of metal crystals would get off to a good start with this nanoporous structure. PVDF-HFP membranes have hydrophobic properties, which make it easy for metal ions to deposit on the surface of the membrane. This is due to the fact that metal salts include water. This hypothesis has two implications: first, it lessens the crystallinity of the polymer; second, it increases the absorbed amount of solution, which could make the surface of the catalyst more accessible to SBH [59]. The use of metal salts for producing nanoscale polymeric NFs has many advantages, including increased electrical properties and gelatinization of the polymer solution, as well as the development of the maximum length of a jet across its axis [66]. This is confirmed by the reduction of NFs size with the addition of metal precursors, which can be clearly observed in Figure 1B–F. SBH reduces Ni and Pd ions in methanol medium to form bimetallic NiPd on the membrane surface. Figure 1B–D,F display SEM images of electrospun Ni@PVDF-HFP, Ni_95_Pd_5_@PVDF-HFP, Ni_90_Pd_10_@PVDF-HFP, Ni_85_Pd_15_@PVDF-HFP, Ni_75_Pd_25_@PVDF-HFP NFs membrane, respectively. It is evident from the images that the synthesized NFs have remarkable nanofibrous architecture with no beads. In addition, the PVDF-HFP NF mats are developed on the nanopores present on its surface; reduced Ni and Pd ions could cover the PVDF-HFP surface. 

**Figure 2:**
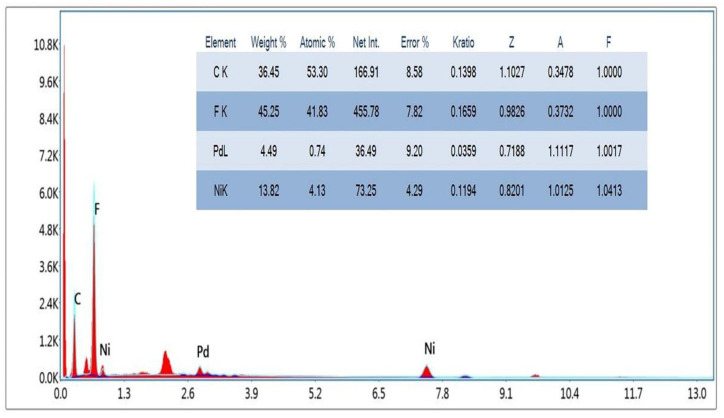
EDX of Ni_75_Pd_25_@PVDF-HFP NFs membrane. The inset shows the percentage weight of the elements.

The EDX of Ni_75_Pd_25_@PVDF-HFP membrane NFs is revealed in Figure 2. It is evident that the chemical makeup of the product comprises of carbon, fluorine, nickel, and palladium elements. The percentage weight of the elements is shown in the inset of Figure 2. 

**Figure 3:**
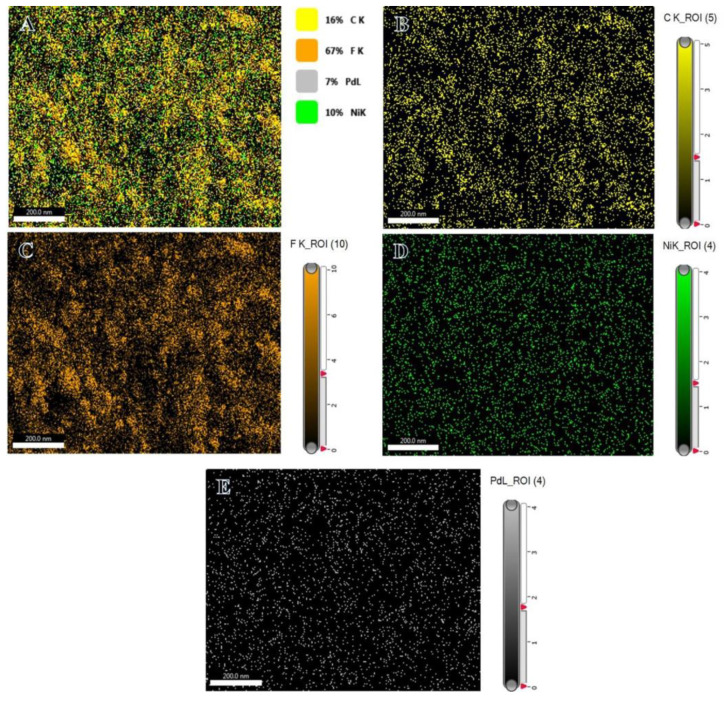
Map showing the allocation of elements (**A**), carbon (**B**), fluorine (**C**), nickel (**D**), and palladium (**E**) in the Ni_75_Pd_25_@PVDF-HFP membranes.

The composition can be clearly observed to be lower than its initial precursors. This may be possibly due to the leaching of a small amount of metal NPs during the reduction and washing processes. The mapping of elements of Ni_75_Pd_25_@PVDF-HFP is displayed in Figure 3. The presence of carbon, fluorine, nickel and palladium is evident from Figure 3B–E. Figure 3A shows that Ni and Pd NPs are widely dispersed over the PVDF-HFP membrane. 

**Figure 4:**
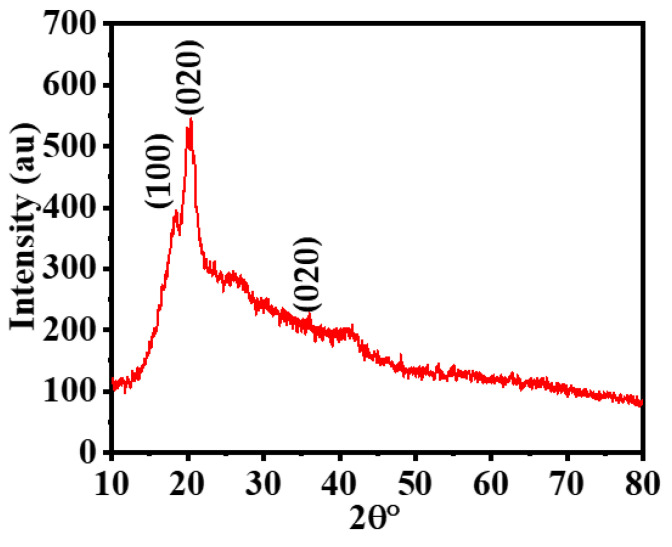
XRD results of Ni_75_Pd_25_@PVDF-HFP NFs membrane.

The XRD pattern of the Ni_75_Pd_25_@PVDF-HFP NFs membrane is displayed in Figure 4. The XRD graph reveals three major diffraction patterns at 2 Ɵ of 18.4°, 20.3°, and 36.01°, which match with the (100), (020), and (021) crystal indices of PVDF-HFP, respectively [67]. However, XRD shows no planes of Ni or Pd NPs because either Pd and Ni NPs are too small or amorphous Ni-Pd formed [26,68,69,70].

**Figure 5:**
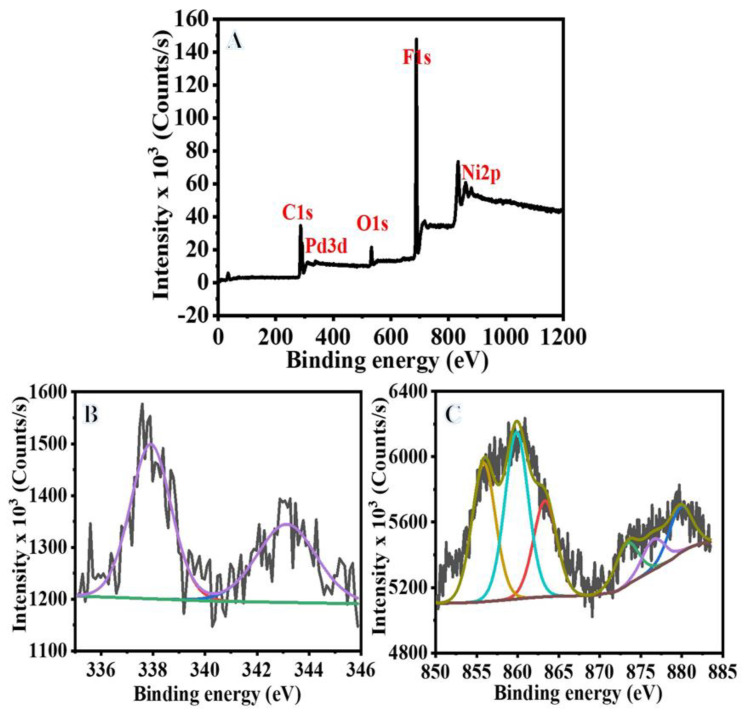
(**A**) Survey spectrum of Ni_75_Pd_25_@PVDF-HFP NFs membrane, (**B**) Pd3d, and (**C**) Ni2p.

The electronic interaction between Pd and Ni is crucial for the catalytic activity of the NiPd. The electron transfer from Ni to Pd in the NiPd@PVDF-HFP membrane NFs may be due to the difference in the electronegativity of Pd (2.20) and Ni (1.92). We employed XPS analysis to examine this aspect [27]. On the full scan XPS spectra, prominent O 1s, C 1s, and F 1s peaks were found at 532.4, 285.08, and 688.9 eV, respectively (see Figure 5A). It is difficult to avoid the presence of thin oxide oxygen due to the exposure of the sample to air and during XPS preparation. Thus, Singh et al., in their study, removed the oxygen film using Ar sputtering before XPS analysis of a Ni/Pd alloy [71]. Figure 5B is an XPS spectra of Pd for the Ni_75_Pd_25_@PVDF-HFP NFs membrane, and it exhibits two peaks at 337.9 and 343.14 eV, both of which are ascribed to metallic Pd since they correspond to the 3d5/2 and 3d3/2 electron-binding energies of Pd, respectively [27]. This demonstrates that most of the Pd in the activated catalyst was reduced during the preparation process. The Ni 2p3/2 and Ni 2p1/2 electron-binding energies were also observed at 853.7 and 872.6 eV, respectively (see Figure 5C); these are also attributed to metallic Ni [71]. Furthermore, Ni shows a high binding energy, which is a result of increasing Pd binding energy and the Ni decreasing electron density [29]. Higher binding energies for Pd [3d5/2] in the bimetallic Ni_75_Pd_25_@PVDF-HFP NFs membrane compared to the monometallic Pd sample are consistent with alloy formation [71]. This change indicates that an electron is being transferred from Ni to Pd, increasing the electron density around Pd atoms, which has been demonstrated to improve H_2_ adsorption and enhance the production of metal-H species, accelerating the SBH dehydrogenation [27]. In other words, Ni and Pd atoms in the Ni_75_Pd_25_@PVDF-HFP NFs membrane catalyst could enhance charge transfer and balancing during the splitting of O-H bonds and B-H bonds in the adsorbed H_2_O and SBH, respectively, to produce H_2_ [29,72,73].

### 3.2. Catalysis Studies

To hydrolyze the substrate effectively, the SBH must quickly penetrate through the support, and the BH_4_^−^ should have access to the NPs’ metal surface. The PVDF-HFP membrane would improve the dispersibility of the NiPd NPs catalyst in H_2_O. The Ni_1−x_Pd_x_ (x = 0–0.3)@PVDF-HFP NF membranes that were synthesized are able to serve as catalysts for the generation of H_2_ from SBH. It is feasible to expect that both Pd and Ni are active phases in the as-prepared NF membranes, as they catalyzed the hydrolysis reaction of SBH [26].

**Figure 6:**
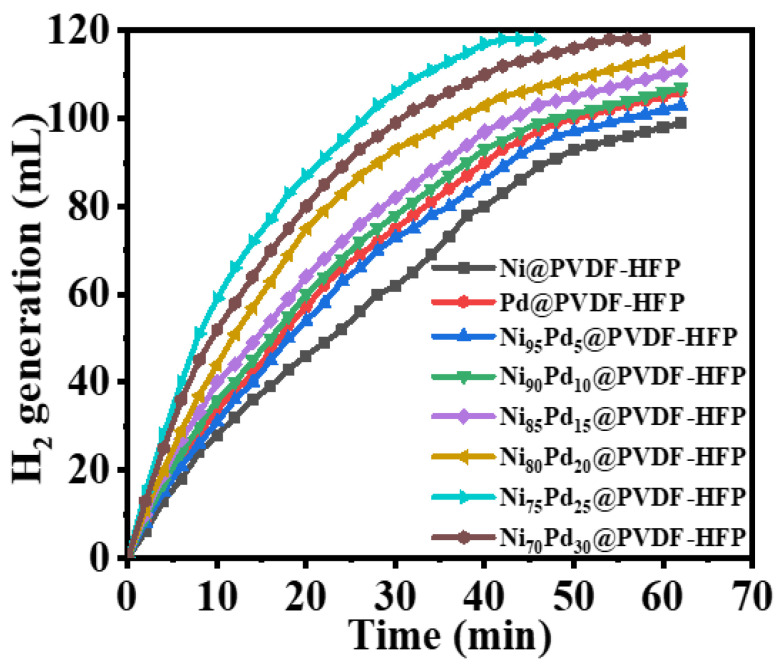
The impact of Ni_1−x_Pd_x_@PVDF-HFP membranes on H_2_ generation by SBH hydrolysis. The catalyst amount = 100 mg, [SBH] = 1 mmol, and T = 298 K.

The catalytic activity of bimetallic catalysts may be improved due to the alloy effect. Catalysts with varying Ni/Pd ratios were used to investigate the effect of Pd concentration on catalytic activity (see Figure 6). Each reaction was performed in an aqueous solution of SBH and catalyst, with strong agitation, at a temperature of 298 K. The time vs. volume of H_2_ produced graphs during the hydrolytic dehydrogenation of SBH in the presence of catalysts with various bimetallic compositions and their counterparts are displayed in Figure 6. The baseline hydrolysis that was carried out using PVDF-HFP NFs demonstrated that the support matrix was inactive because the volume of hydrogen gas produced did not significantly exceed that of the background reaction. It can be inferred from Figure 6 that Pd@PVDF-HFP NFs achieved a relatively higher catalytic activity compared to Ni@PVDF-HFP NFs. The more quickly the hydrolysis is completed, the more active the catalyst will be. Accordingly, the catalytic activity of the bimetallic catalysts is significantly higher than that of their counterparts, which was due to the synergistic effect between the two metals [72,74,75]. Additionally, previous research on Pd/Ni-alloy catalysts in hydrogenation processes revealed that bimetallics with specific molar ratios of Ni/Pd exhibited greater activity than either Pd or Ni monometallic catalysts [26,76,77]. Dopant metals are thought to exert their stimulating effects by increasing the catalyst particles’ active surface area and facilitating electronic interaction between two active metals [78]. Among all the examined NiPd@PVDF-HFP NFs membranes, the Ni_75_Pd_25_@PVDF-HFP NFs membranes demonstrate the maximum catalytic activity with a completion time of 42 min for 1 mmol SBH. Maximum hydrogen production yields, as determined by the hydrolysis of SBH (1 mmol) utilizing the PVDF-HFP NFs catalysts with varying Pd/Ni ratios, are shown in Table 1 along with the respective H_2_ generation rates (k). The rate at which H_2_ is produced does, in fact, increase with the amount of Ni_75_Pd_25_@PVDF-HFP NFs. The activity of the catalyst drops down as Pd concentration is increased. It has been observed that the activity of a catalyst can be increased up to 150% with Pd as compared to the activity achieved with a Ni catalyst. This likely occurs as a result of the synergistic effect between the Ni and Pd [26,71]. The interactions between metal NPs, oxides, and support would make the process of charge transfer easier and expose an increased number of catalytic activation sites [79,80]. Singh et al. demonstrated that the mixture of Ni and Pd nanoparticles (Ni/Pd = 60:40) exhibit poor H_2_ generation activity from hydrous hydrazine [71]. These results indicate that electronic alteration of the catalyst surface and bimetallic phase active sites is essential to generate hydrogen from hydrous hydrazine hydrolysis. Monometallic and alloy catalysts interact differently with reactant molecules [71,81]. Heterometallic bonds with strong metal–metal contacts may improve catalytic efficiency and molecular selectivity compared to monometallic bonds by tailoring the catalyst surface’s bonding pattern to reactant molecules and stabilizing the possible reaction intermediates [71,76,82]. 

**Figure 7:**
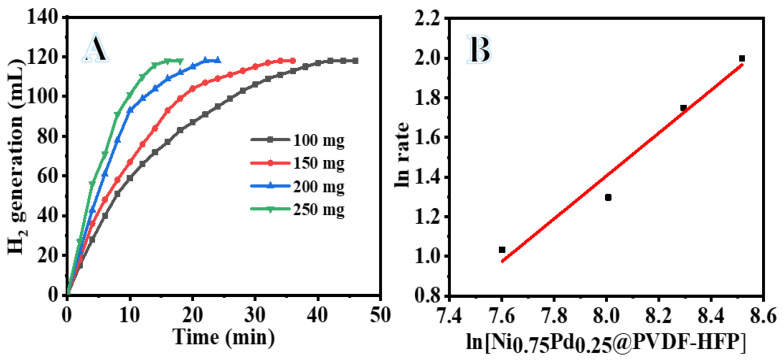
Effect of Ni_75_Pd_25_@PVDF-HFP NFs membrane amount on H_2_ production by SBH hydrolysis (**A**), and H_2_ generation rate vs. amount of catalyst on the logarithmic scale (**B**). [SBH] = 1 mmol and T = 303 K.

Further investigation into the kinetics of the hydrolytic dehydrogenation of SBH by a Ni_75_Pd_25_@PVDF-HFP NFs membrane catalyzed process was carried out by varying the concentrations of the catalysts and SBH, as well as the reaction temperatures. Ni_75_Pd_25_@PVDF-HFP NFs membranes of varying weights (100, 150, 200, and 250 mg) were subjected to hydrolysis. With 100 mg catalyst, the reaction takes 42 min to produce 118 mL of H_2_ (see Figure 7A). The same reactions, when carried out with a higher amount of catalyst, can experience a significantly improved reaction rate, producing hydrogen rapidly. Thus, the reaction takes just 16 min with 250 mg of the catalyst. Table 2 displays the yield and the final volume of hydrogen produced. Hydrogen production rates at various catalyst concentrations were set by adjusting the initial linear regions of the plots, as shown in Figure 7B. The dehydrogenation of SBH follows first-order kinetics with respect to the concentration of Ni_75_Pd_25_@PVDF-HFP NFs membranes, as evident by the nearly linear relationship between the hydrogen production rate and catalyst concentration on the logarithmic scale with a slope of 1.08 (see Figure 7B). On the other hand, under identical conditions, H_2_ can be produced spontaneously from the SBH solution, but only at a rate of 0.47 mL min^−1^ with a yield of just 23.7% in the absence of a catalyst. However, when the catalyst was included, H_2_ production was reliable. Due to this, the catalyst significantly accelerates the process.

**Figure 8:**
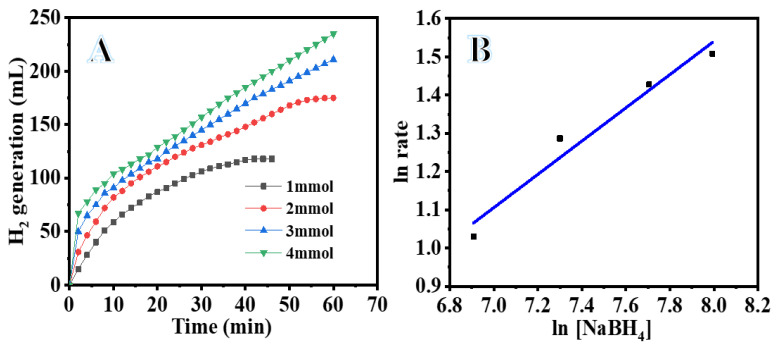
Effect of SBH concentration on H_2_ generation (**A**) and the value of H_2_ generation vs. value of [SBH] on logarithmic scale (**B**). The amount of Ni_75_Pd_25_@PVDF-HFP NFs membrane catalyst = 100 mg and T = 298 K.

In SBH-dependent hydrolysis, the concentration of the catalyst was maintained constant at 100 mg, while the SBH concentrations were varied by 1, 2, 3, and 4 mmol. It is found that the SBH concentration has almost no effect on the production of hydrogen at 298 K (see Figure 8A). We surmise that the Ni_75_Pd_25_@PVDF-HFP NFs membranes catalyzed SBH hydrolysis following zero-order reaction kinetics with respect to the SBH concentration, since the plot of hydrogen generation rate versus SBH concentration on a logarithmic scale (see Figure 8B) corresponds to a line with a slope of 0.43.

**Figure 9:**
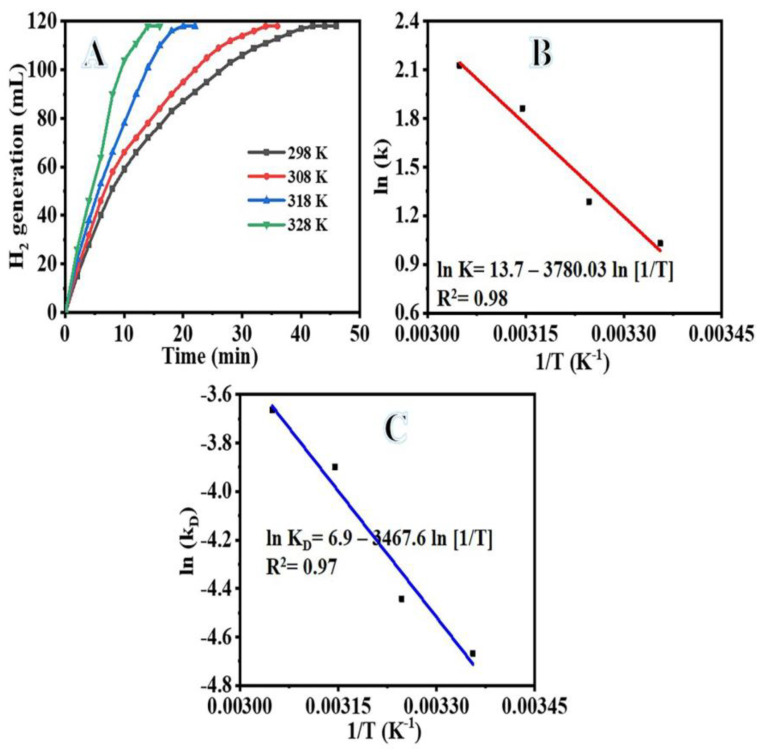
The impact of temperature on the reaction (**A**), ln (k, rate constant) vs. temperature inverse (**B**), and ln KD (K/T) vs. temperature inverse (**C**). The amount of Ni_75_Pd_25_@PVDF-HFP NFs membrane catalyst = 100 mg and [SBH] = 1 mmol.

To examine the effect of temperature on the reaction, 1 mmol of SBH was hydrolyzed employing 100 mg of Ni_75_Pd_25_@PVDF-HFP membranes while the temperature was varied from 298 K to 328 K. The plot of time versus volume of H_2_ produced during SBH hydrolysis at various temperatures is depicted in Figure 9A. Increasing the reaction temperature causes an increase in hydrogen production. An increased reaction temperature improves the catalytic efficiency of Ni_75_Pd_25_@PVDF-HFP NFs membranes for SBH hydrolytic dehydrogenation. At 318 K, the reaction takes just 20 min, compared to 14 min at 328 K. The activation parameters for the hydrolysis processes at different temperatures were determined by fitting the data in Figure 9B,C using Arrhenius and Eyring equations. The rate constants were calculated from the linear part of each plot in Figure 9B by evaluating their slope. The activation energy for the dehydrogenation process was determined to be 31.43 kJ mol^−1^, while ΔH and ΔS were found to be 28.82 kJ mol^−1^ and 0.0576 kJ mol^−1^ K^−1^, respectively. With a negative ΔS, the rate-determining step is likely to use an associative activation mode rather than a dissociative one. Table 3 provides a comparison of the relatively low activation energy of the Ni_75_Pd_25_@PVDF-HFP NFs membranes catalyst compared to the values found in the literature for Ni-based catalysts and Pd-based catalysts. 

**Figure 10:**
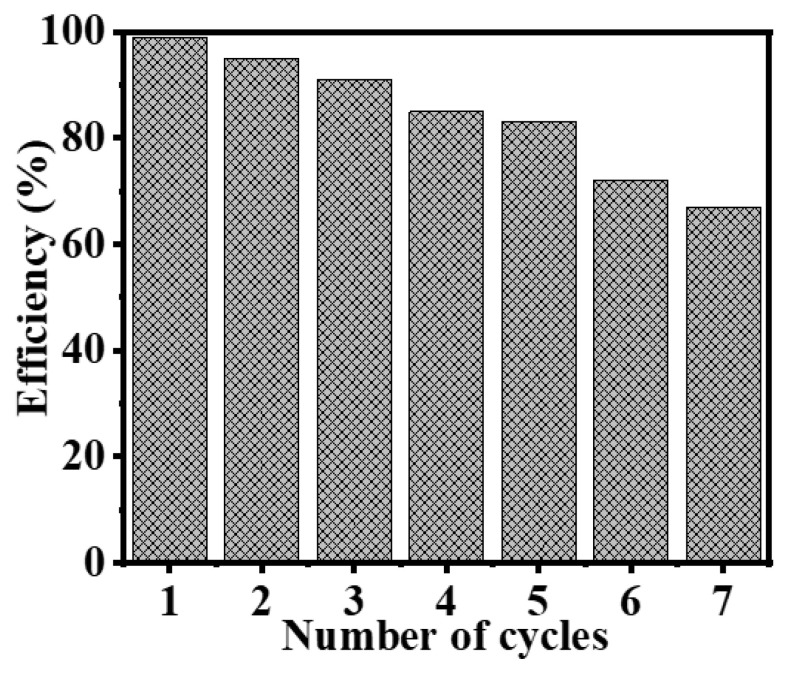
Reusability tests for Ni_75_Pd_25_@PVDF-HFP NFs membrane. The amount of catalyst, [SBH], and T are 100 mg, 1 mmol, and 298 K, respectively.

Moreover, the lifetime of the catalysts utilized in the hydrolysis of SBH was investigated by studying the reusability and recyclability of Ni_75_Pd_25_@PVDF-HFP NFs membranes catalysts (Figure 10). A total of 100 mg of an Ni_75_Pd_25_@PVDF-HFP NFs membranes catalyst was used as a catalyst to hydrolyze 1 mmol of SBH at 298 K. Once the SBH hydrolysis was complete, another 1 mmol of NaBH_4_ was loaded in the previous solution. The Ni_75_Pd_25_@PVDF-HFP NFs membranes catalyst was used without previously being isolated. Once the reaction was complete, the MNFs was simply withdrawn from the catalytic system. A simple separation such as this has several advantages, such as prolonged durability, and service life may be significantly increased, which is a desirable trait for practical applications. The good distribution and immobilization of the metal particles in the MNFs 2-D structure is responsible for the high recyclability of the catalysts. H_2_ production efficiency data indicates a gradual decline in H_2_ generation from the 1st to the 7th cycles of the process. The Ni_75_Pd_25_@PVDF-HFP NFs membranes’ catalytic-efficiency estimates show that after the third, fifth, and seventh runs, the catalyst maintains 85%, 83%, and 67% of its initial activity, respectively. This may have occurred because the reaction products precipitate out onto the membrane’s surface after being reused without any cleaning between the cycles. This prevents the metal active sites from becoming exposed, due to which H_2_ generation slows down. A small decrement is observed in the catalytic performance after the second cycle, which may be attributed to the deposition of the byproduct boron on the Ni_75_Pd_25_@PVDF-HFP surface, and a rise in the viscosity of the solution [83,84,85,86,87,88] can ultimately lead to fewer available active sites or blocked pores.

The SBH hydrolysis kinetic equation catalyzed by a Ni_75_Pd_25_@PVDF-HFP NFs membranes catalyst may be expressed as in Equations (2)–(4), based on the findings of the impacts of catalyst amount, SBH concentration, and reaction temperatures.

(2) $$r=-\frac{d\left[\mathrm{SBH}\right]}{\mathrm{dt}}=k{\left[{\mathrm{Ni}}_{75}{\mathrm{Pd}}_{25}@\mathrm{PVDF-HFP}\right]}^{1.082}{\left[\mathrm{SBH}\right]}^{0.44}$$

(3) $$k={\mathrm{Ae}}^{\left(\frac{-E_{a}}{\mathrm{RT}}\right)}\;\to \;\mathrm{lnk}=\ln\;13.7-\frac{31,430}{8.314T}$$

(4) $$r=-\frac{d\left[\mathrm{SBH}\right]}{\mathrm{dt}}=13.7e^{\left(\frac{-3780}{T}\right)}{\left[{\mathrm{Ni}}_{75}{\mathrm{Pd}}_{25}@\mathrm{PVDF-HFP}\right]}^{1.082}{\left[\mathrm{SBH}\right]}^{0.44}$$

The ΔH and ΔS values can now be used to obtain ΔG using Equations (5) and (6).

(5) $$\ln k_{D}=\ln\frac{k_{B}}{h}+\frac{\Delta S}{R}-\frac{\Delta H}{\mathrm{RT}}$$

(6) ΔG=ΔH−TΔS

The determined values of ΔH and ΔS are as follows: 28.82 kJ mol^−1^ and 0.0576 kJ mol^−1^ K^−1^, respectively, using the Equation (7), which is shown in Figure 9C. The following is a concise summary of the ΔG equation:

(7) ΔG=28.82−0.0576 T

## 4. Conclusions

We successfully prepared bimetallic Ni_1−x_Pd_x_ (x = 0, 0.05, 0.1, 0.15, 0.20, 0.25, 0.3)@PVDF-HFP NFs membranes via two-steps: (1) electrospinning the solution which consists of metallic precursors, and (2) reducing the formed membranes in situ with SBH in methanol media. Bimetallic Ni_1−x_Pd_x_ (x = 0, 0.05, 0.1, 0.15, 0.20, 0.25, 0.3)@PVDF-HFP NFs membranes exhibited high catalytic activity in comparison to their counterparts. The Ni_75_Pd_25_@PVDF-HFP NFs generated the highest volume of H_2_ in a short time in comparison to the other formulations. The kinetics study of the hydrolysis reaction using Ni_75_Pd_25_@PVDF-HFP membranes demonstrated that the reaction is of first order with respect to the amount of the catalyst and zeroth order with respect to SBH concentration, respectively. The obtained value of thermodynamic parameters, namely, Ea, ΔH and ΔS values, were 31.43 kJ mol^−1^, 28.82 kJ mol^−1^ and 0.0576 kJ mol^−1^ K^−1^, respectively. The synthesized NFs are easily separable and reusable, which facilitates their commercialization as hydrogen-storage materials.

## Figures and Tables

**Table 1:** H_2_ evolved yields and rates by polymer membranes used different Ni/Pd ratios at 298 K.

	Ni	Pd	Ni_95_Pd_5_	Ni_90_Pd_10_	Ni_85_Pd1_5_	Ni_80_Pd_20_	Ni_75_Pd_25_	Ni_70_Pd_30_
Volume (mL)	87	78	83	90	94	108	117	101
Yield%	72.5	65	69.2	75	78.3	90	98	84.2
Rate (mL min^−1^)	2.29	2.05	2.18	2.37	2.47	2.84	3.03	2.66

**Table 2:** Effect of Ni_75_Pd_25_@PVDF-HFP amount on the hydrolysis of NaBH_4_.

	Catalyst (gm)			
	0.1	0.15	0.2	0.25
Volume (mL)	118	118	118	118
Yield%	98.3	98.3	98.3	98.3
Reaction time (min)	42	32	22	16
Rate (mL min^−1^)	2.81	3.69	5.36	7.38

**Table 3:** E_a_ of prepared NFs, and catalysts based on Pd and Ni used in H_2_ generation using NaBH_4_.

Catalytic Material	E_a_ (kJ mol^−1^)	Ref.
Ni	42.28	[83]
Ni	71	[84]
Raney Ni	63	[84]
Ni(0)	51.4	[76]
Ni-Ag	16.2	[31]
Pd/C powder	28	[85]
Pd-Ni-B	31.1	[26]
Pd NPs@ [KIT-6]-PEG-imid	35.7	[74]
Ni-hollow PVDF capsules	49.3	[65]
Ni-PVDF hollow fiber	55.3	[86]
([C6(mpy)2][NiCl4]^2−^	56.4	[87]
PVDF-[C6(mpy)2][NiCl4]^2−^	44.6	[88]
NiPd@PVDF-HFP	31.43	This study

## Data Availability

The data presented in this study are available from the corresponding authors upon reasonable request.

## References

[1] Lin F., Zhang A., Zhang J., Yang L., Zhang F., Li R., Dong H. (2021). Hydrogen generation from sodium borohydride hydrolysis promoted by MOF-derived carbon supported cobalt catalysts. Colloids Surf. A Physicochem. Eng. Asp..

[2] Moradi R., Groth K.M. (2019). Hydrogen storage and delivery: Review of the state of the art technologies and risk and reliability analysis. Int. J. Hydrogen Energy.

[3] Abdelhamid H.N. (2021). A review on hydrogen generation from the hydrolysis of sodium borohydride. Int. J. Hydrogen Energy.

[4] Yousef A., Brooks R.M., El-Halwany M., Obaid M., El-Newehy M.H., Al-Deyab S.S., Barakat N.A. (2016). A novel and chemical stable Co–B nanoflakes-like structure supported over titanium dioxide nanofibers used as catalyst for hydrogen generation from ammonia borane complex. Int. J. Hydrogen Energy.

[5] Dinc M., Metin Ö., Özkar S. (2012). Water soluble polymer stabilized iron (0) nanoclusters: A cost-effective and magnetically recoverable catalyst in hydrogen generation from the hydrolysis of sodium borohydride and ammonia borane. Catal. Today.

[6] Demirci U.B. (2017). Ammonia borane, a material with exceptional properties for chemical hydrogen storage. Int. J. Hydrogen Energy.

[7] Tayeh T., Awad A.S., Nakhl M., Zakhour M., Silvain J.F., Bobet J.L. (2014). Production of hydrogen from magnesium hydrides hydrolysis. Int. J. Hydrogen Energy.

[8] Xie X., Ni C., Wang B., Zhang Y., Zhao X., Liu L., Wang B., Du W. (2020). Recent advances in hydrogen generation process via hydrolysis of Mg-based materials: A short review. J. Alloys Compd..

[9] Cai H., Lu P., Dong J. (2016). Robust nickel–polymer nanocomposite particles for hydrogen generation from sodium borohydride. Fuel.

[10] Chen Y., Kim H. (2008). Ni/Ag/silica nanocomposite catalysts for hydrogen generation from hydrolysis of NaBH4 solution. Mater. Lett..

[11] Chen Y., Liu L., Wang Y., Kim H. (2011). Preparation of porous PVDF-NiB capsules as catalytic adsorbents for hydrogen generation from sodium borohydride. Fuel Process. Technol..

[12] Zhang J., Hao J., Ma Q., Li C., Liu Y., Li B., Liu Z. (2017). Polyvinylpyrrolidone stabilized-Ru nanoclusters loaded onto reduced graphene oxide as high active catalyst for hydrogen evolution. J. Nanopart. Res..

[13] Huang Y.-H., Su C.-C., Wang S.-L., Lu M.-C. (2012). Development of Al_2_O_3_ carrier-Ru composite catalyst for hydrogen generation from alkaline NaBH4 hydrolysis. Energy.

[14] Ro G., Hwang D.K., Kim Y. (2019). Hydrogen generation using Pt/Ni bimetallic nanoparticles supported on Fe_3_O_4_@SiO_2_@TiO_2_ multi-shell microspheres. J. Ind. Eng. Chem..

[15] Wu C., Guo J., Zhang J., Zhao Y., Tian J., Isimjan T.T., Yang X. (2019). Palladium nanoclusters decorated partially decomposed porous ZIF-67 polyhedron with ultrahigh catalytic activity and stability on hydrogen generation. Renew. Energy.

[16] Yue C., Yang P., Wang J., Zhao X., Wang Y., Yang L. (2020). Facile synthesis and characterization of nano-Pd loaded NiCo microfibers as stable catalysts for hydrogen generation from sodium borohydride. Chem. Phys. Lett..

[17] Liu S., Chen X., Wu Z.-J., Zheng X.-C., Peng Z.-K., Liu P. (2019). Chitosan-reduced graphene oxide hybrids encapsulated Pd (0) nanocatalysts for H_2_ generation from ammonia borane. Int. J. Hydrogen Energy.

[18] Huff C., Long J.M., Heyman A., Abdel-Fattah T.M. (2018). Palladium nanoparticle multiwalled carbon nanotube composite as catalyst for hydrogen production by the hydrolysis of sodium borohydride. ACS Appl. Energy Mater..

[19] Zhao W., Li W., Lu L., Li F., Zhang H., Zhang S. (2016). Preparation of Colloidal Pd/Ni Bimetallic Nanoparticle Catalysts and Their Catalytic Activity for Hydrogen Generation from Hydrolysis Reaction of Sodium Borohydride. Rare Met. Mater. Eng..

[20] Patel N., Miotello A. (2015). Progress in Co–B related catalyst for hydrogen production by hydrolysis of boron-hydrides: A review and the perspectives to substitute noble metals. Int. J. Hydrogen Energy.

[21] Dönmez F., Ayas N. (2021). Synthesis of Ni/TiO_2_ catalyst by sol-gel method for hydrogen production from sodium borohydride. Int. J. Hydrogen Energy.

[22] Kiren B., Ayas N. (2022). Nickel modified dolomite in the hydrogen generation from sodium borohydride hydrolysis. Int. J. Hydrogen Energy.

[23] Kassem A.A., Abdelhamid H.N., Fouad D.M., Ibrahim S.A. (2019). Metal-organic frameworks (MOFs) and MOFs-derived CuO@ C for hydrogen generation from sodium borohydride. Int. J. Hydrogen Energy.

[24] Hashimi A.S., Nohan M.A.N.M., Chin S.X., Khiew P.S., Zakaria S., Chia C.H. (2020). Copper nanowires as highly efficient and recyclable catalyst for rapid hydrogen generation from hydrolysis of sodium borohydride. Nanomaterials.

[25] Liao J., Huang H. (2020). Magnetic sensitive Hericium erinaceus residue chitin/Cu hydrogel nanocomposites for H2 generation by catalyzing NaBH4 hydrolysis. Carbohydr. Polym..

[26] Liu W., Cai H., Lu P., Xu Q., Zhongfu Y., Dong J. (2013). Polymer hydrogel supported Pd–Ni–B nanoclusters as robust catalysts for hydrogen production from hydrolysis of sodium borohydride. Int. J. Hydrogen Energy.

[27] Du Y., Wang K., Zhai Q., Chen A., Xi Z., Yan J., Kang X., Chen M., Yuan X., Zhu M. (2018). Alloyed palladium-nickel hollow nanospheres with interatomic charge polarization for improved hydrolytic dehydrogenation of ammonia borane. Int. J. Hydrogen Energy.

[28] Zhang J., Dong Y., Liu Q., Zhou M., Mi G., Du X. (2019). Hierarchically alloyed Pd–Cu microarchitecture with tunable shapes: Morphological engineering, and catalysis for hydrogen evolution reaction of ammonia borane. Int. J. Hydrogen Energy.

[29] Cai H.-K., Jiang Z.-Y., Xu S., Xu Y., Lu P., Dong J. (2022). Polymer Hydrogel Supported Ni/Pd Alloys for Hydrogen Gas Production from Hydrolysis of Dimethylamine Borane with a Long Recyclable Lifetime. Polymers.

[30] Wang W., Zhou W., Li W., Xiong X., Wang Y., Cheng K., Kang J., Zhang Q., Wang Y. (2020). In-situ confinement of ultrasmall palladium nanoparticles in silicalite-1 for methane combustion with excellent activity and hydrothermal stability. Appl. Catal. B Environ..

[31] Al-Thabaiti S.A., Khan Z., Malik M.A. (2019). Bimetallic Ag-Ni nanoparticles as an effective catalyst for hydrogen generation from hydrolysis of sodium borohydride. Int. J. Hydrogen Energy.

[32] Hosseini M.G., Daneshvari-Esfahlan V., Wolf S., Hacker V. (2021). Novel Bimetallic Pd–X (X= Ni, Co) Nanoparticles Assembled on N-Doped Reduced Graphene Oxide as an Anode Catalyst for Highly Efficient Direct Sodium Borohydride–Hydrogen Peroxide Fuel Cells. ACS Appl. Energy Mater..

[33] Ye K., Ma X., Cang R., Wang G., Cheng K., Wang G., Cao D. (2017). Nickel nanowires decorated with ultra-low palladium loading as an effective electrocatalyst for NaBH 4 oxidation. Catal. Sci. Technol..

[34] Huang W., Xu F., Liu X. (2021). Superior hydrogen generation from sodium borohydride hydrolysis catalyzed by the bimetallic Co–Ru/C nanocomposite. Int. J. Hydrogen Energy.

[35] Paksoy A., Kurtoğlu S.F., Dizaji A.K., Altıntaş Z., Khoshsima S., Uzun A., Balcı Ö. (2021). Nanocrystalline cobalt–nickel–boron (metal boride) catalysts for efficient hydrogen production from the hydrolysis of sodium borohydride. Int. J. Hydrogen Energy.

[36] Chen C.-W., Chen C.-Y., Huang Y.-H. (2009). Method of preparing Ru-immobilized polymer-supported catalyst for hydrogen generation from NaBH4 solution. Int. J. Hydrogen Energy.

[37] Metin Ö., Özkar S. (2008). Synthesis and characterization of poly (*N*-vinyl-2-pyrrolidone)-stabilized water-soluble nickel (0) nanoclusters as catalyst for hydrogen generation from the hydrolysis of sodium borohydride. J. Mol. Catal. A Chem..

[38] Metin O., Ozkar S. (2009). Hydrogen generation from the hydrolysis of ammonia-borane and sodium borohydride using water-soluble polymer-stabilized cobalt (0) nanoclusters catalyst. Energy Fuels.

[39] Metin Ö., Şahin Ş., Özkar S. (2009). Water-soluble poly (4-styrenesulfonic acid-co-maleic acid) stabilized ruthenium (0) and palladium (0) nanoclusters as highly active catalysts in hydrogen generation from the hydrolysis of ammonia–borane. Int. J. Hydrogen Energy.

[40] Malvadkar N.A., Sekeroglu K., Dressick W.J., Demirel M.C. (2011). Catalytic activity of cobalt on nanotextured polymer films for hydrogen production. J. Power Sources.

[41] Rakap M., Özkar S. (2009). Intrazeolite cobalt (0) nanoclusters as low-cost and reusable catalyst for hydrogen generation from the hydrolysis of sodium borohydride. Appl. Catal. B Environ..

[42] Zahmakiran M., Özkar S. (2008). Intrazeolite ruthenium (0) nanoclusters: A superb catalyst for the hydrogenation of benzene and the hydrolysis of sodium borohydride. Langmuir.

[43] Zahmakiran M., Özkar S. (2009). Zeolite-confined ruthenium (0) nanoclusters catalyst: Record catalytic activity, reusability, and lifetime in hydrogen generation from the hydrolysis of sodium borohydride. Langmuir.

[44] Saka C., Eygi M.S., Balbay A. (2020). CoB doped acid modified zeolite catalyst for enhanced hydrogen release from sodium borohydride hydrolysis. Int. J. Hydrogen Energy.

[45] Abdelhamid H.N. (2021). Dehydrogenation of sodium borohydride using cobalt embedded zeolitic imidazolate frameworks. J. Solid State Chem..

[46] Abutaleb A., Zouli N., El-Halwany M., Ubaidullah M., Yousef A. (2021). Graphitic nanofibers supported NiMn bimetallic nanoalloys as catalysts for H2 generation from ammonia borane. Int. J. Hydrogen Energy.

[47] Brooks R., Maafa I.M., Al-Enizi A., El-Halwany M., Ubaidullah M., Yousef A. (2019). Electrospun bimetallic nicr nanoparticles@ carbon nanofibers as an efficient catalyst for hydrogen generation from ammonia borane. Nanomaterials.

[48] Al-Enizi A.M., Nafady A., El-Halwany M., Brooks R.M., Abutaleb A., Yousef A. (2019). Electrospun carbon nanofiber-encapsulated NiS nanoparticles as an efficient catalyst for hydrogen production from hydrolysis of sodium borohydride. Int. J. Hydrogen Energy.

[49] Li T., Xiang C., Chu H., Xu F., Sun L., Zou Y., Zhang J. (2022). Catalytic effect of highly dispersed ultrafine Ru nanoparticles on a TiO_2_-Ti_3_C_2_ support: Hydrolysis of sodium borohydride for H_2_ generation. J. Alloys Compd..

[50] Yousef A., Barakat N.A., Khalil K.A., Unnithan A.R., Panthi G., Pant B., Kim H.Y. (2012). Photocatalytic release of hydrogen from ammonia borane-complex using Ni (0)-doped TiO_2_/C electrospun nanofibers. Colloids Surf. A Physicochem. Eng. Asp..

[51] Erat N., Bozkurt G., Özer A. (2022). Co/CuO–NiO–Al_2_O_3_ catalyst for hydrogen generation from hydrolysis of NaBH_4_. Int. J. Hydrogen Energy.

[52] Kılınç D., Şahi Ö., Saka C. (2018). Salicylaldimine-Ni complex supported on Al_2_O_3_: Highly efficient catalyst for hydrogen production from hydrolysis of sodium borohydride. Int. J. Hydrogen Energy.

[53] Dai P., Zhao X., Xu D., Wang C., Tao X., Liu X., Gao J. (2019). Preparation, characterization, and properties of Pt/Al_2_O_3_/cordierite monolith catalyst for hydrogen generation from hydrolysis of sodium borohydride in a flow reactor. Int. J. Hydrogen Energy.

[54] Aydın K., Kulaklı B.N., Filiz B.C., Alligier D., Demirci U.B., Figen A.K. (2020). Closing the hydrogen cycle with the couple sodium borohydride-methanol, via the formation of sodium tetramethoxyborate and sodium metaborate. Int. J. Energy Res..

[55] Lo C.T., Karan K., Davis B.R. (2007). Kinetic studies of reaction between sodium borohydride and methanol, water, and their mixtures. Ind. Eng. Chem. Res..

[56] Zhang H., Xu G., Zhang L., Wang W., Miao W., Chen K., Cheng L., Li Y., Han S. (2020). Ultrafine cobalt nanoparticles supported on carbon nanospheres for hydrolysis of sodium borohydride. J. Renew. Energy.

[57] Al-Enizi A.M., Yousef A., Shaikh S.F., Pandit B., El-Halwany M.M. (2022). Electrospun Nickel Nanoparticles@ Poly (vinylidene fluoride-hexafluoropropylene) Nanofibers as Effective and Reusable Catalyst for H_2_ Generation from Sodium Borohydride. Arab. J. Chem..

[58] Raghavan P., Zhao X., Kim J.K., Manuel J., Chauhan G.S., Ahn J.H., Nah C. (2008). Ionic conductivity and electrochemical properties of nanocomposite polymer electrolytes based on electrospun poly (vinylidene fluoride-co-hexafluoropropylene) with nano-sized ceramic fillers. Electrochim. Acta.

[59] Mališ J., Mazúr P., Schauer J., Paidar M., Bouzek K. (2013). Polymer-supported 1-butyl-3-methylimidazolium trifluoromethanesulfonate and 1-ethylimidazolium trifluoromethanesulfonate as electrolytes for the high temperature PEM-type fuel cell. Int. J. Hydrogen Energy.

[60] Vijayakumar E., Subramania A., Fei Z., Dyson P.J. (2015). High-performance dye-sensitized solar cell based on an electrospun poly(vinylidene fluoride-co-hexafluoropropylene)/cobalt sulfide nanocomposite membrane electrolyte. RSC Adv..

[61] Zhang P., Li R., Huang J., Liu B., Zhou M., Wen B., Xia Y., Okada S. (2021). Flexible poly(vinylidene fluoride-co-hexafluoropropylene)-based gel polymer electrolyte for high-performance lithium-ion batteries. RSC Adv..

[62] Tian X., Jiang X. (2008). Poly(vinylidene fluoride-co-hexafluoropropene) (PVDF-HFP) membranes for ethyl acetate removal from water. J. Hazard. Mater..

[63] Zhang Y., Zhao Y., Bakenov Z., Gosselink D., Chen P. (2014). Poly (vinylidene fluoride-co-hexafluoropropylene)/poly (methylmethacrylate)/nanoclay composite gel polymer electrolyte for lithium/sulfur batteries. J. Solid State Electrochem..

[64] Chen Y., Shi Y., Wang Y. (2015). Preparation of hollow poly (vinylidene fluoride) capsules containing nickel catalyst for hydrogen storage and production. Int. J. Energy Res..

[65] Kang H.-C., Chen Y., Arthur E.E., Kim H. (2014). Microstructural control of catalyst-loaded PVDF microcapsule membrane for hydrogen generation by NaBH4 hydrolysis. Int. J. Hydrogen Energy.

[66] Stephan A.M., Nahm K.S., Kulandainathan M.A., Ravi G., Wilson J.J. (2006). Poly (vinylidene fluoride-hexafluoropropylene)(PVdF-HFP) based composite electrolytes for lithium batteries. Eur. Polym. J..

[67] Liu Y., Zhang J., Guan H., Zhao Y., Yang J.-H., Zhang B. (2018). Preparation of bimetallic Cu-Co nanocatalysts on poly (diallyldimethylammonium chloride) functionalized halloysite nanotubes for hydrolytic dehydrogenation of ammonia borane. Appl. Surf. Sci..

[68] Yao Q., Lu Z.-H., Wang Y., Chen X., Feng G. (2015). Synergetic catalysis of non-noble bimetallic Cu–Co nanoparticles embedded in SiO_2_ nanospheres in hydrolytic dehydrogenation of ammonia borane. J. Phys. Chem. C.

[69] Subramanian N.D., Balaji G., Kumar C.S.S.R., Spivey J.J. (2009). Development of cobalt–copper nanoparticles as catalysts for higher alcohol synthesis from syngas. Catal. Today.

[70] Singh S.K., Iizuka Y., Xu Q. (2011). Nickel-palladium nanoparticle catalyzed hydrogen generation from hydrous hydrazine for chemical hydrogen storage. Int. J. Hydrogen Energy.

[71] Zhou Y.H., Zhang Z., Wang S., Williams N., Cheng Y., Luo S., Gu J. (2018). rGO supported PdNi-CeO_2_ nanocomposite as an efficient catalyst for hydrogen evolution from the hydrolysis of NH_3_BH_3_. Int. J. Hydrogen Energy.

[72] Fang R., Yang Z., Wang Z., Ran J., Yan Y., Zhang L. (2022). Novel non-noble metal catalyst with high efficiency and synergetic photocatalytic hydrolysis of ammonia borane and mechanism investigation. Energy.

[73] Al-shaikh H., Lasri J., Knight J.G., Al-Goul S.T. (2022). Palladium mesoporous nanoparticles Pd NPs@[KIT-6] and Pd NPs@[KIT-6]-PEG-imid as efficient heterogeneous catalysts for H_2_ production from NaBH4 hydrolysis. Fuel.

[74] Singh A.K., Xu Q. (2013). Synergistic catalysis over bimetallic alloy nanoparticles. ChemCatChem.

[75] Lu P., Teranishi T., Asakura K., Miyake M., Toshima N. (1999). Polymer-protected Ni/Pd bimetallic nano-clusters: Preparation, characterization and catalysis for hydrogenation of nitrobenzene. J. Phys. Chem. B.

[76] Fuku K., Sakano T., Kamegawa T., Mori K., Yamashita H. (2012). Enhanced hydrogenation activity of nano-sized Pd–Ni bimetal particles on Ti-containing mesoporous silica prepared by a photo-assisted deposition method. J. Mater. Chem..

[77] Patel N., Fernandes R., Miotello A. (2010). Promoting effect of transition metal-doped Co–B alloy catalysts for hydrogen production by hydrolysis of alkaline NaBH4 solution. J. Catal..

[78] Tonbul Y., Akbayrak S., Özkar S. (2016). Palladium (0) nanoparticles supported on ceria: Highly active and reusable catalyst in hydrogen generation from the hydrolysis of ammonia borane. Int. J. Hydrogen Energy.

[79] Yen H., Seo Y., Kaliaguine S., Kleitz F. (2015). Role of metal–support interactions, particle size, and metal–metal synergy in CuNi nanocatalysts for H2 generation. ACS Catal..

[80] Rodriguez J.A., Goodman D.W. (1992). The nature of the metal-metal bond in bimetallic surfaces. Science.

[81] Yamaguchi A., Hiyoshi N., Sato O., Osada M., Shirai M. (2010). Lignin gasification over charcoal-supported palladium and nickel bimetal catalysts in supercritical water. Chem. Lett..

[82] Ozay O., Aktas N., Inger E., Sahiner N. (2011). Hydrogel assisted nickel nanoparticle synthesis and their use in hydrogen production from sodium boron hydride. Int. J. Hydrogen Energy.

[83] Kaufman C.M., Sen B. (1985). Hydrogen generation by hydrolysis of sodium tetrahydroborate: Effects of acids and transition metals and their salts. J. Chem. Soc. Dalton Trans..

[84] Patel N., Patton B., Zanchetta C., Fernandes R., Guella G., Kale A., Miotello A. (2008). Pd-C powder and thin film catalysts for hydrogen production by hydrolysis of sodium borohydride. Int. J. Hydrogen Energy.

[85] Chen Y., Shi Y., Liu X., Zhang Y.J. (2015). Preparation of polyvinylidene fluoride–nickel hollow fiber catalytic membranes for hydrogen generation from sodium borohydride. Fuel.

[86] Chinnappan A., Kim H., Baskar C., Hwang I.T. (2012). Hydrogen generation from the hydrolysis of sodium borohydride with new pyridinium dicationic salts containing transition metal complexes. Int. J. Hydrogen Energy.

[87] Chinnappan A., Kim H. (2012). Nanocatalyst: Electrospun nanofibers of PVDF–Dicationic tetrachloronickelate (II) anion and their effect on hydrogen generation from the hydrolysis of sodium borohydride. Int. J. Hydrogen Energy.

[88] Yang K., Yao Q., Huang W., Chen X., Lu Z.-H. (2017). Enhanced catalytic activity of NiM (M = Cr, Mo, W) nanoparticles for hydrogen evolution from ammonia borane and hydrazine borane. Int. J. Hydrogen Energy.

